# Incidence of Surgical Site Infection and Factors Associated among Cesarean Deliveries in Selected Government Hospitals in Addis Ababa, Ethiopia, 2019

**DOI:** 10.1155/2020/9714640

**Published:** 2020-02-22

**Authors:** Hana Lijaemiro, Semarya Berhe Lemlem, Jembere Tesfaye Deressa

**Affiliations:** Addis Ababa University College of Health Sciences, Department of Midwifery, Addis Ababa, Ethiopia

## Abstract

**Background:**

One-third to two-thirds of operated patients in low-income countries acquire surgical site infection, which is nine times higher when compared to high-resource countries. Identifying the incidence and risk factors that contribute to surgical site infection following cesarean delivery is a step ahead for preventing and reducing the problem. Nonetheless, the distribution of the problem in Addis Ababa, where the rate of cesarean delivery is relatively high compared to other parts of the country, is under investigation.

**Objective:**

The aim of this study is to assess the incidence of surgical site infection among cesarean deliveries and factors associated with it in selected governmental hospitals found in Addis Ababa, Ethiopia, in 2019.

**Method:**

A hospital-based prospective cohort study design was employed to follow 175 women, who gave birth by cesarean delivery in selected government hospitals in Addis Ababa, from March 11 to April 9, 2019. Convenience sampling method was used to select study units from the randomly selected hospitals. Descriptive statistics were run for determining the rate of cesarean delivery surgical site infection. Presence and degree of association between outcome and independent variables were computed through bivariate logistic regression analysis and factors that had *p* < 0.2 significance level in the bivariate logistic regression analysis were considered in the multivariable logistic regression analysis.

**Result:**

From 166 participants who completed 30-day follow-up, 25 (15%) of the participants developed surgical site infection. Age, gestational age, duration of operation, and ≥5 vaginal examinations showed a significant association with the outcome variable with AOR (95% CI) of ((AOR = 1.504, 95% CI: (1.170 – 1.933, *p* < 0.2 significance level in the bivariate logistic regression analysis were considered in the multivariable logistic regression analysis. *p* < 0.2 significance level in the bivariate logistic regression analysis were considered in the multivariable logistic regression analysis. *p* < 0.2 significance level in the bivariate logistic regression analysis were considered in the multivariable logistic regression analysis. *p* < 0.2 significance level in the bivariate logistic regression analysis were considered in the multivariable logistic regression analysis. *Conclusion and recommendation*. Surgical site infection rate is higher and certain associations lost due to small sample size. Further interventional studies with vast sample size are recommended.

## 1. Introduction

Cesarean delivery, often known as cesarean section, is an operative delivery of a fetus via maternal abdominal and uterine wall incision [1]. Cesarean delivery is indicated in cases of antepartum hemorrhage and fetal distress, and CD becomes mandatory to prevent potential maternal and neonatal morbidity and mortality [2].

Medically indicated cesarean section has a pronounced influence to prevent life-threatening conditions, such as obstetric fistula and birth asphyxia, and save maternal and child life [3]. However, CD also accompanies the risk of infection, hemorrhage, and uterine rupture and placentation problems in current and future pregnancies [3–6].

Hence, in 1985, World Health Organization (WHO) declared that the optimal threshold for cesarean section rate should be 10–15% based on the data available especially from Northern Europe, where perinatal morbidities and mortalities were low with the above rate [7]. This declaration aims at enhancing the practice of performing CD for those who need it and avoid the procedure for medically ineligible clients. Even though this declaration is debatable to date, CD rate exceeding 15–20% is not yet associated with improved perinatal conditions [8].

Nonetheless, recent studies revealed that the rate of cesarean delivery is rising unpredictably leading to actual, potential, and lifelong maternal and neonatal complications. Half of the cesarean section procedures exceeding the WHO maximum CD rate threshold are performed in Brazil and China [8]. This can be evidenced by a 40.5% CD rate exhibited in Latin America and the Caribbean region [9]. On the contrary, the poor perinatal maternal, neonatal, and infant outcomes in low-resource countries with less than 5% CD rate may attribute to the socioeconomic situation in the countries [10]. One of the short-term morbidities which take place after cesarean section is surgical site infection (SSI). It is a postsurgery infection in parts of the body where it is performed. The Center for Disease Control and Prevention (CDC)'s National Nosocomial Infection Surveillance System serves as a de facto standard for defining and classifying SSI. It classifies SSI as incisional and organ/space SSI. Incisional SSI, in turn, has two divisions: superficial and deep SSI. The infection on both types of incisional SSIs is restrained to the incision site only. Superficial SSI involves skin and subcutaneous tissue of the incisional site, while deep SSI distributes to the muscle and fascia of the incisional site. If the infection spreads to body wall layers that are not manipulated during the surgical procedure, it is named as organ (space) SSI [11].

The leading healthcare-associated infection (HAI) in low-income countries is SSI affecting one-third to two-thirds of operated patients, which is nine times higher when it is compared to high-resource countries [12]. A study conducted in 25 low- and middle-income African countries also witnessed most complications following surgery, and 10.2% were due to surgical site infection [13]. It is also undeniable that SSI is the second most frequently reported HAI in even high-income countries such as USA and Europe [14].

The majority of the conditions leading to HAIs including SSIs are at ease of intervention. Factors such as poor waste disposal and environmental hygiene practice reduced standard precaution implementation trend and development of local procedures and guidelines for each clinical activity is feasible to be intervened by intrainstitution and national health organization divisions [12, 14]. Governmental involvement might be necessary in fulfilling infrastructure and essential equipment for the procedure and recruiting an adequate number of staff with appropriate knowledge and skill [12]. Host-related characteristics such as advanced maternal age, raised BMI, and coexisting maternal disease might influence CD SSI. Likewise, adverse conditions surrounding pregnancy and CD would have an effect on maternal CD SSI status.

## 2. Methods and Materials

### 2.1. Study Setting and Period

The study was conducted from March 11 to May 24, 2019, in Addis Ababa, Ethiopia. The city was divided into 10 subcities which has 14 governmental hospitals in 2019 according to a data from Ethiopian Ministry of Health.

### 2.2. Study Design

A 30-day hospital-based prospective cohort follow-up of women who gave birth by CD in the first month of the data collection period was conducted.

### 2.3. Source Population

The source population was drawn from women who delivered by CD in government hospitals in Addis Ababa.

### 2.4. Study Population

The sample population was drawn from women who delivered by CD in the first 30 days of the data collection period in the selected four governmental hospitals in Addis Ababa, Ethiopia.

### 2.5. Inclusion Criteria

Women who delivered by CD in the study period and were willing to participate in the study, who had a permanent address for reporting their condition, and who were mentally fit to differentiate and report their status were included in the study.

### 2.6. Exclusion Criteria

Women who were severely ill and died during the first phase of the study period were excluded.

### 2.7. Sampling Methods

Among 14 public hospitals in Addis Ababa, 4 were selected by simple random sampling method. Then, based on the number of cesarean deliveries in the hospital, a proportionate number of study units were allocated for each hospital. All CDs that took place during the first phase of data collection time were included as study unit until the allocated sample size was attained (convenience sampling method).

### 2.8. Sample Size Determination

Single population proportion formula was utilized to calculate the sample size and adding 10% contingency sample size became 165, *p* = estimation of CD rate (11% in a study done in Hawassa Teaching and Referral Hospital [15]).

### 2.9. Data Collection

#### 2.9.1. Data Collection Procedure

All mothers who delivered by CD in selected government hospitals during the study period were asked for their willingness to participate in the study. The medical record of those volunteer women was reviewed for the presence of SSI risk factors. Each mother was interviewed through telephone for the development of SSI syndromes within 30 postoperative days.

#### 2.9.2. Data Collection Tool

The data collection tool was adapted from a study done in Zimbabwe and modified based on the contextual situation. It contains questions that assess maternal characteristics such as age, gravidity, parity, BMI, coexisting morbidities; peripartum maternal conditions such as maternal health condition, PROM, number of vaginal examinations, trial of labor before CD; and procedural characteristics such as duration of operation, type of surgery, type of anesthesia, qualification of the surgeon, type and timing of antibiotics administration, preoperative skin preparation, and the likes.

#### 2.9.3. Data Analysis

The collected data were coded, entered, checked, and cleaned by Epi-data version 3.1 and were exported to SPSS version 20, for data analysis. The proportion of CD SSI was computed by running descriptive statistics, followed by bivariate and multivariable logistic regression to determine the statistical association between independent and dependent variables. Presence and degree of association between outcome and independent variables were computed through odds ratio with 95% confidence interval (CI) and *p* value < 0.05.

#### 2.9.4. Data Quality Control

Data collectors were trained on the objective of the study, the data collection procedures, and the data collection tool. The data collection tool was adapted from a study done in Zimbabwe and modified based on contextual situations. The tool was pretested in 10% of similar population in another Specialized Referral Hospital to assure the validity of it and modify accordingly.

Collected data were reviewed and checked for completeness and consistency on a daily basis.

## 3. Result

A 30-day post-CD prospective follow-up through telephone for development of syndromes of SSI was conducted from April 22 to May 24, 2019, for 175 mothers who gave birth by CD in four selected government hospitals in Addis Ababa from March 11 to April 19, 2019. Among 175 mothers, 166 (approximately 95%) of them completed the 30-day telephone interview, while 11 (5 from TASH, 1 from ZMGH, and 3 form Y12MCGH) of them were lost before completing the follow-up.

### 3.1. Sociodemographic and Obstetrics Characteristics of Participants

The age of the participants in the study ranged from 19 to 40 years, while majority of the participants (approximately 75%) were less than 30 years old. In addition to this, around 95% of the study participants were from urban. Most of the women involved in the study had 3 or less parity status (93.4%); among this, Para I, Para II, and Para III account for 53.6%, 24.1%, and 15.7% of the participants, respectively.

Minimal number of the participants, 28.9%, was found to have previous CD, suggesting most CDs in this study were mostly due to other obstetric emergencies. Women with prolonged PROM also account for only 15.7% of the participants as presented below (Table 1).

### 3.2. Operation-Related Characteristics of Participants

#### 3.2.1. Indication for CD and Related Comorbidities Identified

Majority of the participants (86%) in the study were referred from other facilities. Among them, the most common indication for CD in the study period was fetal distress (40.4%), followed by CPD (19.9%) and arrest of labour (13.3%), while the rest is accountable for other indications such as PIH and oligohydramnios.

Since the sample size is small (*n* = 166), adequate representatives of the comorbidities hypertension (30.7%), DM (4.8%), and HIV (3.6%) were not available during the study period.

#### 3.2.2. Preoperative Preparation

Preoperative antibiotics prophylaxis was provided for around 92% of the participants, and among them, 66.3% of the participants gained 2 g ampicillin as a prophylaxis while the rest utilized 1 g ceftriaxone for the same purpose.

Most of the health professional's handwashing (70.5%) before CD was conducted utilizing plain soap and water, which was available in most setups. Skin preparation for CD of all participants was done by alcohol, while iodine was utilized in addition to alcohol in approximately 20% of the participants.

As depicted in Table 2, only 59 (35.5%) of the study participants were not clipping of the pubic hair. Among those who clipping of their pubic hair, 78 (40%) were shiny on top at their home, while the rest 28 (17%) and 1 (0.6%) were lacking hair at preoperative wards and on the operating table, respectively.

### 3.3. Operative and Postoperative Characteristics

Only 36 (21.7%) operations were conducted electively without any medical or other emergence (Table 3).

Residents perform majority of CDs (94.6%) followed by specialists (4.8%) and GP (general physicians) (0.6%) during the study period. The most prevalent type of skin incision used throughout this study was transverse incision being performed in about 161 (97%) of the study participants. CDs were completed with a range of 4 to 70 minutes; the median time to complete the operation is 26.5 minutes. Skin closure after CD was determined by the physician's choice, which was interrupted in 21 (12.7%) of the study participants.

Post-CD antibiotics were provided for 156 (94%) of the participants. Two grams of ampicillin IV was used in 70% of those who received the antibiotics. Besides, all study participants did not develop PPH during the study period.

### 3.4. Incidence Rate of SSI

**Figure 1:**
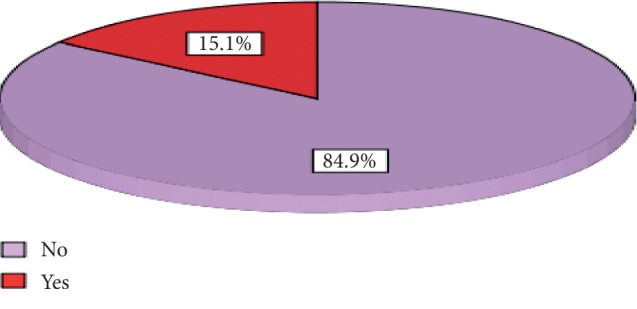
Incidence rate of SSI among women who delivered by CD in four governmental hospitals in Addis Ababa, Ethiopia, 2019.

From 166 participants who completed 30-day follow-up, 25 (15%) of the participants developed SSI depicted below (Figure 1). Among them, 17 (68%) developed superficial SSI that only required outpatient wound dressing and use of broad-spectrum antibiotics. But 8 (32%) developed deep SSI that required prolonged hospital stay.

All mothers (166) in this study were followed for 30 days starting from the first postoperative day. All post-CD SSIs were detected during the 30-day telephone interview, i.e., no SSI was detected during the in-patient stay. Moreover, fifteen (60%) of the SSIs were detected from days 11–17 followed by days 1–10 which exhibits 9 (36%) of SSIs and days 25–30 that enables detection of 1 SSI.

#### 3.4.1. Factors Associated with Cesarean Delivery among SSI

Bivariate logistic regression was run for variables in this study. Variables that scored a *p* value of less than or equal to 0.2 were considered in multivariable logistic regression.

In the multivariate analysis, age, gestational age, and duration of operation showed a significant association with a *p* value of ≤0.05. According to the latter analysis, every one year increment in age leads to 1.5 times greater risk ((AOR = 1.504, 95% CI: (1.170–1.933))) to develop SSI. Similarly, every one-minute increment in the duration of operation has 1.1 times higher risk ((AOR = 1.108, 95% CI: (1.025–1.197)) for having SSI.

Giving birth by CD at term was also found to be 98.1% protective ((AOR = 0.019, 95% CI: (0.001–0.291))), for the incidence of SSI compared to that of postterm deliveries. Nonetheless, association could not be made for that of preterm delivery in this study presented in Table 4.

## 4. Discussion

One hundred seventy-five women who delivered by CD in the selected hospitals in the first month (phase) of the data collection were weekly interviewed through telephone for the development of SSI syndromes for four consecutive weeks, taking the date of operation as day 1. Among them, nine (approximately 5%) participants were lost before completing their follow-up, and therefore, they were excluded from the study.

Post-CD SSI was detected in twenty-five (15.1%) of the participants. From this, one can appreciate the use of postdischarge infection surveillance for immediate evaluation and improvement of CD service, since post-CD in-patient stay is decreasing from time to time. The rate, however, might be underestimated due to a significant number of lost participants.

The rate, 15.1%, is significantly high compared to the results of studies done in Polish hospital, US academic institution, Israel health institution, Thai-Myanmar border hospital, a hospital in Oman, two hospitals in Libya, three sub-Saharan African countries, and Tanzania which showed an incidence rate of 0.5%, 5.5%, 3.7%, 6.2%, 2.6%, 2.53–3.07%, 7.6%, and 10.9%, respectively [6, 16–23]. This difference might be due to a difference in SSI definition, distribution of risk factors among the studied group, study time, socioeconomic status, and healthcare delivery system.

Even though most of the studies reviewed in this study utilized the definition of CDC for SSI diagnosis, some, like the study in Libya, utilized more specific criteria to diagnose the issue unlike this study. For example, Libya's study utilized healing progress and the presence of certain bacteria to diagnose the condition in addition to the CDC's criteria. This might contribute to the wide difference in the incidence rate of post-CD SSI between Libya's and the present study [18].

It can also be noticed that compared to previous studies conducted in Ethiopia, the incidence rate of post-CD SSI in this study is high and this can be exemplified by the rates of 6.8% in Lemlem-Carl Hospital, 11% in Hawassa, and 11.7% in Ayder Hospital [15, 21, 24]. The difference might be due to the study design, study place, and sample size.

However, the rate is in line with a study conducted in Jordan that finds out a 14.4% incidence of post-CD SSI [25]. This might partially be due to the proximity of the study time and means of data collection used by both studies was also similar. Some of the variables considered in this study show a strong association with the outcome variable. A one-year increment in age exhibits 1.5 times greater risk ((AOR = 1.504, 95% CI: (1.170–1.933))) to develop post-CD SSI. This result is in line with findings of studies done in Israel and Libya, which showed age increment is a risk factor for post-CD SSI [17, 18]. This agreement might be due to reduced ability of cell growth and repair which is inevitable as age increases, and furthermore, gradual changes in endocrine and immune system with aging might also contribute.

The finding, however, contradicts that of a study performed in three sub-Saharan African countries that demonstrated younger age was associated with a higher incidence of SSI [22]. This might be due to overrepresentation of young age population and the endemic nature of the condition.

The study also showed giving birth by CD at term was 98.1% protective ((AOR = 0.019, 95% CI: (0.001–0.291))) for the incidence of SSI compared to that of postterm deliveries. Even though direct association between preterm delivery and the outcome variable could not be found in this study, it agrees with studies in the USA and Israel that labouring at term can protect infection by avoiding complications of preterm labour such as hastened labour [16, 17].

This study revealed a one-minute increment in the duration of operation has 1.1 times higher risk ((AOR = 1.108, 95% CI: (1.025–1.197))) for having SSI. The finding was in line with a study done in Lemlem-Karl Hospital, Ethiopia [24]. This might be due to the extended time of operation that leads to more tissue damage and rise in the introduction of various microorganisms into the peritoneal cavity.

## 5. Conclusion

Rate of incidence of post-CD SSI was high in this study. The variables PROM, length of labour trial, type of operation, indication of operation, type of incision, and postoperative antibiotics were not significant in this study. However, they should be tested in a study with larger sample size which has an adequate representation of the cases.

Maternal age, gestational age, and duration of operation were found to have a strong association with the study variable.

## Figures and Tables

**Table 1:** Sociodemographic and obstetric characteristics of study participants (*n* = 166) who gave birth through CD in four selected government hospitals in Addis Ababa, Ethiopia, 2019.

Variables	Category	Post-CD SSI				Total (%)
		Yes		No		
		*N*	%	*N*	%	
Age	<30 yrs	4	3.3	120	96.7	124 (74.7%)
	≥30 yrs	21	50	21	50	42 (25.3%)
Residence	Urban	24	15.2	134	84.8	158 (95.2%)
	Rural	1	12.5	7	87.5	8 (4.8%)
History of previous CD	Yes	13	27.1	35	72.9	48 (28.9%)
	No	12	10.2	106	89.8	118 (71.1%)
Parity	Para I	11	12.4	78	87.6	89 (53.6%)
	Para II	7	17.5	33	82.5	40 (24%)
	Para III	5	19.2	21	80.8	26 (15.7%)
	Para IV	1	10	9	90	10 (6%)
	Para V	1	100	0	0	1 (0.6%)
PROM	No rupture	10	11.4	78	88.6	88 (53%)
	≤12 hrs	4	7.7	48	92.3	52 (31.3%)
	≥12 hrs	11	42.3	15	57.7	26 (15.7%)

**Table 2:** CD indication, related comorbidities, and preoperative characteristics of study participants (*n* = 166) in four selected government hospitals in Addis Ababa, Ethiopia, 2019.

Variables	Category	Post-CD SSI				Total (%)
		Yes		No		
		*N*	%	*N*	%	
CPD	Yes	10	30.3	23	69.7	33 (19.9%)
	No	15	11.3	118	88.7	133 (80.1%)
Fetal distress	Yes	8	11.9	59	88.1	67 (40.4%)
	No	17	17.2	82	82.8	99 (59.6%)
Arrest of labour	Yes	3	13.6	19	86.4	22 (13.3%)
	No	22	15.3	122	84.7	144 (86.7%)
Hypertension	Chronic HTN	2	14.3	12	85.7	14 (8.4%)
	PIH	10	27	27	73	37 (22.3%)
	No HTN	13	11.3	102	88.7	115 (69.3%)
DM	Chronic DM	0	0	2	100	2 (1.2%)
	Gestational DM	1	16.7	5	83.3	6 (3.6%)
	No DM	24	15.2	134	84.8	158 (95.2%)
HIV	Detected before pregnancy	0	0	3	100	3 (1.8%)
	Detected during pregnancy	0	0	3	100	3 (1.8%)
	No HIV detected	25	15.6	135	84.4	160 (96.4%)
Referral status	Yes	20	14	123	86	143 (86.1%)
	No	5	21.7	18	78.3	23 (13.9%)
Antibiotics prophylaxis	Yes	23	15	130	85	153 (92.2%)
	No	2	15.4	11	84.6	13 (7.8%)
Plain soap and water use	Yes	20	14.6	117	85.4	137 (82.5%)
	No	5	17.2	24	82.8%	29 (17.5%)
Antimicrobial soap and water use	Yes	5	17.2	24	82.8	29 (17.5%)
	No	20	14.6	117	85.4	137 (82.5%)
Client's pubic hair shiny on top	Yes	16	15	91	85	107 (64.5%)
	No	9	15.3	50	84.7	59 (35.5%)
Place pubic hair bald	Ward	4	14.3	24	85.7	28 (16.7%)
	Operating table	0	0	1	100	1 (0.6%)
	Home	12	15.4	66	84.6	78 (45%)

**Table 3:** Operative and postoperative characteristics of study participants (*n* = 166) among CDs in four selected government hospitals in Addis Ababa, Ethiopia, 2019.

Variables	Category	Post-CD SSI				Total (%)
		Yes		No		
		*N*	%	*N*	%	
Type of operation	Elective	1	2.8	35	97.2	36 (21.7%)
	Emergency	24	18.7	106	81.5	130 (78.3%)
CD performed by	GP	0	0	1	100	1 (0.6%)
	Resident	23	14.65	134	85.4	157 (94.6%)
	Specialist	2	25	6	75	8 (4.8%)
Skin incision	Transverse	22	13.7	139	86.3	161 (97%)
	Vertical	3	60	2	40	5 (3%)
Skin closure	Interrupted	2	9.5	19	90.5	21 (12.7%)
	Continuous	23	15.9	122	84.1	145 (87.3%)
Post-CD antibiotics	Yes	20	12.8	136	87.2	156 (94%)
	No	5	50	5	50	10 (6%)

**Table 4:** Factors associated with post-CD infection among women (*n* = 166) who gave birth by CD in four selected government hospitals in Addis Ababa, Ethiopia, from March 11 to May 24, 2019.

Variable	Category	SSI		COR (95% CI)	*p* value	AOR (95% CI)	*p* value
		Yes	No				
Age	<30 yrs	4	120	1		1	0.01
	≥30 yrs	21	21	1.44 (1.010–2.023)	0.030	1.5 (1.170–1.933)	
PROM	No	10	78	1		1	
	≤12 hrs	4	48	0.65 (0.193–2.188)	0.121	0.235 (0.02–2.808)	0.35
	>12 hrs	11	15	5.72 (2.064–15.85)^*∗*^	^0.142^	3.982 (0.381–41.614)	0.27
Number of vaginal examinations	No	2	54	1		1	
	1–4	5	36	2.454 (0.552–10.916)	0.472	10.732 (0.438–263.006)	0.34
	≥5	17	52	5.776 (1.597–20.89)^*∗*^	0.132	13.076 (1.018–168.002)	0.048
Length of labour trial before CD	≤24 hrs	10	108	1		1	
	>24 hrs	15	33	4.909 (2.016–11.955)^*∗*^	0.113	3.408 (0.402–28.897)	0.232
Gestational age	<37 wks	3	26	0.238 (0.063–0.897)^*∗*^	0.131	0.074 (0.004–1.315)	0.32
	37–40 wks	5	80	0.129 (0.044–0.376)^*∗*^	0.151	0.019 (0.001–0.291)^*∗∗*^	0.004
	>40 wks	17	35	1		1	
Type of operation	Elective	1	35	1		1	
	Emergency	24	106	7.925 (1.034–60.734)^*∗*^	0.145	7.667 (0.190–309.384)	0.243
CPD	Yes	10	23	3.420 (1.368–8.552)^*∗*^	0.010	1.860 (0.158–21.857)	0.120
	No	15	118	1		1	
Type of skin incision	Transverse	22	139	1		1	
	Vertical	3	2	9.477 (1.498–59.964)^*∗*^	0.010	0.046 (0.001–2.408)	0.253
Postoperative antibiotics	Yes	20	136	1		1	
	No	5	5	6.800 (1.807–25.595)^*∗*^	0.142	8.999 (0.037–213.220)	0.254
Surgical wound class	Class I	10	78	1		1	
	Class II	4	48	0.65 (0.193–2.188)	0.246	0.235 (0.02–2.808)	0.132
	Class III	11	55	5.72 (2.064–15.85)^*∗*^	0.132	3.982 (0.381–41.614)	0.148

^*∗*^ *p* value ≤ 0.2 in bivariate analysis; ^*∗∗*^*p* value <0.05 in multivariable analysis.

## Data Availability

All data generated or analyzed during the study were included in this published article and its additional information files. The raw data and materials are available and can be obtained from Addis Ababa University School of Nursing and Midwifery Research and Publication Committee, Ethiopia, and from the corresponding author on reasonable request.

## Supplementary Materials

Supplementary Materials Appendix: data collection tool: Post-cesarean delivery surgical site infection data collection tool.
