# Supplementary material for: Incidence of Surgical Site Infection and Factors Associated among Cesarean Deliveries in Selected Government Hospitals in Addis Ababa, Ethiopia, 2019
Source: Obstet Gynecol Int. 2020 Feb 22;2020:9714640. doi: 10.1155/2020/9714640 (PMC7057000; doi:10.1155/2020/9714640)
Supplement: Supplementary Materials — Appendix: data collection tool: Post-cesarean delivery surgical site infection data collection tool. [file 9714640.f1.docx]

## Appendix: Data Collection Tool

| **Post-cesarean delivery surgical site infection data collection tool** | | | | | | | |
| --- | --- | --- | --- | --- | --- | --- | --- |
| 1 | Participant code: _____ Age: ____ Weight: ____ Kg. Height: ____cm.  Gravidity: _______ Parity: ______ | | | | | | |
|  | Hospital: ______________________ Ward: ______________ | | | | | | |
|  | Date of admission: __________ Participant contact number: ______________  Is the client referred from another facility: □ Yes □ No | | | | | | |
| 2 | Underlying conditions:  Diabetes mellitus□ Yes before pregnancy □ Yes during pregnancy □No Hypertension□ Yes before pregnancy □ Yes during pregnancy □ No  Cardiac failure □ Yes before pregnancy □ Yes during pregnancy □ No  HIV status□ Positive before pregnancy □ Positive during pregnancy □Negative  If HIV positive, is client on ART□ Yes before pregnancy □ Yes during pregnancy □ No  Is patient on anti-TB treatment□ Yes before pregnancy □ Yes during pregnancy □ No Other, specify__________________ | | | | | | |
| 3 | Gestational age □ <37 weeks □ 37 – 40weeks □ > 40 weeks | | | | | | |
| 4 | Duration that client was in labour□ no labour □ < 24 hours  □ > 24 hours □ No record  Number of vaginal examinations before CD: □ No vaginal examination  □ 1 – 4 vaginal examinations  □ ≥ 5 vaginal examinations | | | | | | |
| 5 | Length of time membranes ruptured prior to cesarean delivery  □ No rupture□ >12 hours  □ <12 hours □ No records | | | | | | |
| 6 | Date of operation ______________ Time of operation _______________  Duration of operation ___________ Start time _________ End time ___________  Type of operation □ Elective □ Emergency  Type of skin incision □ Transverse □ Vertical  History of previous cesarean section □ Yes □ No  Type of anesthesia provided □ General □ Local □ Spinal □ Epidural  The operation is performed by □ Student □ GP □ Resident □ Specialist | | | | | | |
| 7 | Does antibiotic prophylaxis provided □ Yes □ No  If yes, state type and dose of antibiotics given ____________________________  Time prophylactic antibiotics is given □ Exact time _________________  If exact time is not available, tick the appropriate one :  □ < 15 min. □ 15 – 30 min.□ 30 – 45 min. □ 45 – 60 min □ > 1 hour | | | | | | |
| 8 | State solution used for surgeon’s hand preparation:  □ Plain soap and water □Antimicrobial soap and water □Other specify………  Antiseptic used for peri operative skin preparation  □Aqueous butadiene □2%Chlohexidine/Alcohol □Other specify……… | | | | | | |
| 9 | Was client shaved? □ Yes □ No; If yes, state where?  □Ward □Anesthetic room□ On the operating table □ Home | | | | | | |
| 10 | Surgical wound classification for caesarean section  Please tick one:  □Class I: Clean  ► Caesarean Section, elective, no pre-rupture of membranes or trial of labour  □Class II: Clean Contaminated  ►Caesarean Section, emergency involving pre-rupture of membranes less than 12hours and /or trial of labour  □Class III:Contaminated  ► Rupture of membranes more than 24hours  □Class IV: Dirty  ►Purulent amniotic fluid | | | | | | |
| 11 | Skin closure: □Interrupted sutures□ Continuous | | | | | | |
| 12 | Post-partum hemorrhage □ Yes □ No | | | | | | |
| 13 | **Post op findings** | **Day 0** | **Day 1** | **Day 2** | **Day 3** | **Day 4** | **Day 5** |
|  | Temperature |  |  |  |  |  |  |
|  | Pulse |  |  |  |  |  |  |
|  | Day sutures removed |  |  |  |  |  |  |
| 14 | **Antibiotic and dose**  **Given** | **Day 0** | **Day 1** | **Day 2** | **Day 3** | **Day 4** | **Day 5** |
|  |  |  |  |  |  |  |  |
| 15 | State presence of any of the following infection symptoms during inpatient stay:  Purulent drainage from the incision □ Yes □ No  Wound dehiscence □ Yes □ No  Presence of at least one of the following signs or symptoms of infection   - - - - Pain or tenderness at operation site□       - Localized swelling□       - Redness□       - Fever (>38°C) □       - Hotness of skin□ | | | | | | |
| 16 | Date of onset of symptoms: ……………………  Date patient discharged………………………  Results of wound swab, if any: Organisms isolated:………………………………………………  Antibiotic Susceptibility: Sensitive to: ………………………………………………………  Resistant to: ……………………………………………………………………………………… | | | | | | |
| **17. Post Discharge Surveillance** | | | | | | | |
| **Review Week 1: Post Discharge Day 4-10**  Is patient experiencing any of the following  infection symptoms:  Pain/tenderness at operation site: □Yes □No  Purulent discharge at wound site□Yes □No  Wound dehiscence □Yes □No  Localized swelling □Yes □No  Redness □Yes □No  Hotness of skin □Yes □No  Date of onset of symptoms………………  Outcome ……………………………… | | | | **Review week 2: Post discharge Day 11 – 17**  Is patient experiencing any of the following  infection symptoms:  Pain/tenderness at operation site: □Yes □No  Purulent discharge at wound site □Yes □No  Wound dehiscence □Yes □No  Localized swelling □Yes □No  Redness □Yes □No  Hotness of skin □Yes □No  Date of onset of symptoms………………  Outcome ……………………………… | | | |
| **Review week 3: Post discharge Day 18 – 24**  Is patient experiencing any of the following  infection symptoms:  Pain/tenderness at operation site: □Yes □No  Purulent discharge at wound site □Yes □No  Wound dehiscence □Yes □No  Localized swelling □Yes □No  Redness □Yes □No  Hotness of skin □Yes □No  Date of onset of symptoms………………  Outcome ……………………………… | | | | **Review week 4: Post discharge Day 25 - 30**  Is patient experiencing any of the following  infection symptoms:  Pain/tenderness at operation site: □Yes □No  Purulent discharge at wound site □Yes □No  Wound dehiscence □Yes □No  Localized swelling □Yes □No  Redness □Yes □No  Hotness of skin □Yes □No  Date of onset of symptoms………………  Outcome ……………………………… | | | |
| **18**. SSI detected: □Yes □No  If Yes state when SSI was detected:□ During admission□Post discharge  Date SSI detected: ………………………  Type of SSI:□ Superficial □ Deep □ Organ/Space | | | | | | | |

Thank you
